# Systematic Review of Focal and Salvage Cryotherapy for Prostate Cancer

**DOI:** 10.7759/cureus.26400

**Published:** 2022-06-28

**Authors:** Yew Fung Chin, Naing Lynn

**Affiliations:** 1 Urology, Anglia and Ruskin University, Cambridge, GBR; 2 Urology, Royal Shrewsbury Hospital, Shrewsbury, GBR

**Keywords:** complication, survival, salvage, primary, prostate cancer, cryotherapy

## Abstract

Cryotherapy is one of the recognised ablative modalities for both primary and salvage therapy for prostate cancer. It presents an alternative, less invasive treatment for an organ-confined disease, improved preservation of surrounding tissue and a more suitable option for patients who are unfit for radical prostatectomy. Nevertheless, the currently available literature is relatively too scarce to provide definite conclusions regarding the treatment outcomes in cryotherapy. The present study aimed to review current oncological and survival outcomes in cryotherapy for primary and recurrent prostate cancer. Furthermore, this study aimed to establish the complications and functional outcomes of cryotherapy for prostate cancer. A literature search was performed on the PubMed, Cochrane and Google Scholar databases. Current guidelines and recommendations from the European Association of Urology were also reviewed. The search keywords used included ‘Cryotherapy, Prostate Cancer’, ‘Cryoablation, Prostate Cancer’ and ’Cryosurgery, Focal Prostate Cancer’. Truncations and Boolean operators were used with the keywords. All relevant studies from after 2015, including abstracts and non-English research assessing oncological and functional outcomes and complications, were included. Twenty-six studies consisting of 11,228 patients were reviewed. Fifteen studies assessed the outcomes of primary cryotherapy, whereas 11 studies reported the outcomes in salvage therapy. The patient's age ranged 55-85 years, and the pre-procedural prostate-specific antigen (PSA) ranged 0.01-49.33 ng/mL. A total of 2031 patients were classified to be at low risk, 2,995 were at moderate risk and 253 were at high risk on the D’Amico prostate cancer risk classification system. Follow-ups ranged from 9.0 to 297.6 months. The disease-specific survival rate was 65.5%-100.0%, overall survival was 61.3%-99.1%, the PSA nadir was 0.01-2.63 ng/mL and the overall biochemical recurrence rate was 15.4%-62.0%. The complications included erectile dysfunction (3.7%-88.0%), urinary retention (2.13%-25.30%) and bladder neck stricture/stenosis (3.0%-16.7%). The functional assessment showed a mixture of improved, unchanged or worsened post-procedural outcomes in primary therapy. This systematic review did not find significant differences in the cancer-specific, overall and biochemical-free survival rate between the primary and salvage cryotherapy cohorts. The most common complications encountered in both cohorts were erectile dysfunction, urinary incontinence, lower urinary tract/bladder neck stricture and infection. More prospective and double-arm studies are critically needed to provide guidance on the careful selection of patient cohorts for cryotherapy, whether for curative or salvage intent.

## Introduction and background

Background

Disease Prevalence

According to Cancer Research UK, prostate cancer is the most common malignancy in the UK male population, consisting of 26% of all new cancer diagnoses in 2017 [1]. For the past 20 years, the diagnosis rate has gradually increased, with an increased detection rate of up to 41% between 1993 and 2017. This is attributed to the introduction of the easily accessible test for prostate-specific antigen (PSA), a plasmatic glycoprotein that is commonly released by a normal prostate gland. A high PSA level correlating with a patient clinical context would prompt further investigation for prostate cancer [2]. Increased education and awareness of prostate cancer in the community have remarkably increased the detection rate of prostate cancer [3]. Moreover, cancers detected early are organ-confined diseases, which have significantly reduced the diagnosis of metastasis [4,5].

Unlike other malignancies, prostate cancer is relatively unique because its natural progression is more protracted [6]. It is relatively prevalent, with up to 50% of the male population in the 70-79 years age group likely having histologically abnormal/cancerous but previously undiagnosed prostate cancer diagnosed by autopsy [7].

Cancer Diagnosis and Primary Treatment

To avoid over-detection and unnecessary treatment for clinically insignificant prostate cancer, the D’Amico risk stratification model was established (D’Amico 1998) (Table 1) [8,9]. The stratification system considers the PSA level, biopsy results (Gleason scoring) and radiologic staging, usually pelvic magnetic resonance imaging, to differentiate between clinically significant and insignificant diseases and decide whether further treatment is indicated.

**Table 1:** D’amico Risk stratification Source: Gomella et al.

	PSA ng/mL	Gleason Sum	Clinical Staging
Low Risk	<10	<6	
Intermediate risk	10 to 20	7	T2b
High risk	>20	8 to 10	>T2c

For clinically insignificant or low-risk disease, conservative management, such as watchful waiting or active surveillance involving regular PSA monitoring and repeated annual biopsy, has been recognised to be one of the treatment options for this particular group, with the active surveillance guidelines recommended by the National Institute for Health and Care Excellence (NICE) in Table 2 [10]. This is further supported by the recent ProtecT trial study, which found no significant difference in 10 years of cancer-specific survival among three cohorts of patients with low- or intermediate-risk prostate cancer under active surveillance, external beam radiotherapy and radical prostatectomy [11]. Nevertheless, a conservative approach to active surveillance has been related to anxiety and the psychological burden with disease progression [12]. On top of this, regular follow-up/biopsy is associated with potential secondary complications, such as risk of post-procedural sepsis, urethral or rectal bleeding and acute urinary retention, which also impact patient outcomes [13].

**Table 2:** NICE: Active Surveillance

Timing	Test
Beginning of Active Surveillance (AS)	Multiparametric MRI if not performed
At Year 1 of AS	Every 3-4 months: Measure PSA level
	Monitoring of PSA Kinetics
	Every 6-12 months: DRE
	At 12 months prostate Biopsy
Year 2-4 of AS	Every 3-6 months: Measure PSA
	Monitoring of PSA Kinetics
	Every 6-12 months: DRE
Year 5 and beyond of AS	Every 6 months: Measure PSA
	Monitoring of PSA Kinetics
	Every 12 months of DRE

Conventional prostate cancer treatment, including radiotherapy and radical prostatectomy, has been considered the gold standards against localised prostate cancer. In radical prostatectomy, remarkable advancements in terms of techniques and technologies, from open prostatectomy to robotic-assisted laparoscopic prostatectomy, have improved immediate and post-operative outcomes and complications. Bahn et al. [14] have shown that radical prostatectomy provided long-term efficacy with a 15 year disease-specific mortality of only 4%-7%. Common post-operative complications included erectile dysfunction and urinary incontinence. Despite introducing techniques for nerve-sparing injury, cases of functionality reduction persisted. Other complications included bladder neck stricture/stenosis, post-operative bleeding, infection, bladder injury and incomplete resection of tumour requiring further treatment [15,16].

Radiotherapy is commonly administered either as external beam therapy or brachytherapy. Radiation therapy has a mortality rate of only 3%-6% [17]. Nonetheless, both treatments have significant effects on patient quality of life and a high risk of post-operative complications. Furthermore, radical prostatectomy is considered a major surgery, which is not suitable for all patients, especially cohorts with multiple co-morbidities (history of cardiovascular or cardiopulmonary disease, diabetes mellitus, previous pelvic surgery, radiotherapy etc.), which would further increase the risk of post-surgical complications.

Background on Cryotherapy

Cryotherapy was introduced as alternative ablative therapy for prostate cancer, which induces tissue destruction via intercellular ice crystal formation, subsequent intracellular dehydration, cell shrinkage and intracellular ice formation, triggering cell membrane disruption and hence apoptosis. Cryotherapy also induces acute pH changes via immediate freezing and thawing. Thawing initiates a necrosis cascade, especially within microvasculature endothelial cells, causing extensive oedema and inflammation, thereby severing the blood supply to cancer cells, a process identified as ‘coagulative necrosis’. The necrosis process would, in turn, exhibit extracellular effects by triggering an inflammatory cascade in the surrounding cells [18]. Cryotherapy is administered at a variety of freezing temperature doses (ranging from −20°C to −60°C) and frequencies of conducting the freeze-thaw cycle. According to the recommendation by the American Urology Association, the optimum freezing temperature is −40°C for 3 min per cycle to achieve tumour eradication [19,20]. In other studies, −40°C is the recommended target temperature for achieving a ‘lethal dose’ via the formation of intracellular ice crystals to induce cellular damage [18,21].

Cryotherapy was first introduced in the 1960s as an experimental treatment for localised prostate cancer [18]. First-generation cryotherapy was utilised without imaging guidance and using a larger-size probe. These features led to a high incidence of complications with urethral and rectal damage, limiting the application of cryotherapy for the next two decades. In fact, the use of cryotherapy was halted at one point because of the high risk of complications with unproven clinical outcomes. The interest in cryosurgery has since peaked again because of improvements in cryo-ablative technologies with the introduction of guiding mechanisms using both magnetic resonance imaging (MRI) and ultrasound (Gage 2007). The application of a smaller vacuum-insulated probe of 2.4 mm and multi-parametric MRI fusion images improved the precision targeting of lesions while preserving surrounding tissues [22]. Moreover, urethral warming through a catheter has reduced the effects of tissue sloughing during cryotherapy. Other improvements featured in current third-generation cryo-technologies include the use of gas-based conduction using argon gas for immediate freezing and helium for thawing.

Despite the variations in equipment and monitoring setups between urological centres, the technical principle of achieving prostate tissue ablation with monitoring would be similar. Currently, the common setup uses trans-rectal ultrasonography to determine the position of the cryoablation probe, which is inserted into prostate tissue through the trans-perineal approach. The probe is installed with a thermal-coupling needle to manage the target temperature. The reading will be monitored together with geometric readings for indications of the freezing and thawing cycles throughout the procedure. For urethral and rectal protection from frost injury, urethral warming catheters and saline injection within Douglas’ pouch are used. During the procedure, ultrasound imaging is also used to allow the operator to visualise the effect of freezing via the formation of ice balls within the target location and thus avoid affecting other essential structures, such as the rectal wall and urethral and prostatic neurovascular bundles [17, 21,22].

In terms of target population, most studies have focused on patients with low and intermediate D’Amico risks, including a Gleason score/grade group (GG) less than 8/GG4, radiological staging less than pT2b and PSA level less than 20 ng/mL [23-25]. Some study series have attempted to also include high-risk localised diseases, showing promising results in disease control and minimal complications [26-29]. A primary limitation of cryotherapy is its dependence on the prostate size, especially for primary cryotherapy. Most studies were limited to prostate volumes below 60 mL. In cases with sizes 60-90 mL, a patient would be offered androgen deprivation therapy (ADT) to shrink the prostate size prior to commencing therapy [30].

Oncological treatment outcomes are usually assessed via repeat measurement of serum PSA post-treatment. During the Radiation Therapy Oncology Group-American Society for Therapeutic Radiology and Oncology (ASTRO) Consensus Conference in 2006, the Phoenix definition of biochemical failure, which is defined as an increase of 2 ng/mL or more from the PSA nadir, was identified as the cut-off for determining treatment failure/biochemical recurrence [31]. Nevertheless, this definition is based on patients who underwent radiotherapy; hence, it must be interpreted cautiously for other prostate cancer treatment modalities. However, many recent cryotherapy case studies still used the Phoenix definition in measuring oncological outcomes. Barqawi et al. evaluated patients who met the Phoenix definition of biochemical recurrence and found that only 40% of the subgroup cohort had positive repeat biopsies, which questions the suitability of PSA monitoring for indicating recurrence. Other means of assessing patient oncological outcomes include cancer-specific survival, overall survival, PSA nadir level and time required until indication for salvage therapy indicated or biochemical recurrence [32]. Because of the heterogeneity of reporting cryotherapy oncological and survival outcomes, updated systematic reviews and analyses are warranted.

Focal vs. Whole-Gland Therapy

Newer generations of cryotherapy technology and the utilisation of multi-parametric MRI fusion imaging have improved the identification and treatment of target lesions. Promising alternative treatments, including focal treatment using modalities such as cryotherapy and high-intensity focused ultrasound (HIFU), have been reported with better oncological and functional outcomes. For the treatment of localised or locally advanced prostate cancer, a paradigm shift has been observed in treatment coverage, from the initial whole-gland cryotherapy to hemi-gland therapy and now targeted focal therapy [33]. Hugh et al. have proposed that the index lesion of prostate cancer is primarily affecting prognosis. Focal therapy presents an ideal and intermediate solution in between conservative management and radical treatment, the latter of which can lead to overtreatment, thereby severely affecting functionality and quality of life. Moreover, the minimally invasive nature of the procedure provides an alternate option for patients who are not suitable for surgery. However, focal therapy may potentially miss lesions, which can lead to seeding and disease progression. Early studies have shown that targeted focal therapy is an appealing alternative to focally ablate cancerous tissue while preserving as much functionality as possible without compromising life expectancy due to recurrence or disease progression.

Salvage Therapy

According to Agarwal et al, one-third of patients with localised prostate cancer who underwent primary treatment have a risk of biochemical recurrence [34]. Current evidence and guidance have provided definite follow-up pathways for biochemical recurrence after radical prostatectomy (NICE 2019, European Association of Urology [EAU] 2019). The evidence for salvage cryotherapy remains scarce, whereas traditional salvage radical prostatectomy after primary radiotherapy has been associated with a high risk of complications. Almost one-third of recurrences consisted of localised recurrence; hence, focal salvage treatment is another potential alternative to reduce the risks associated with further salvage radical therapy. Common salvage therapy options for recurrence of localised cancer are ADT, radiotherapy, salvage prostatectomy, cryotherapy, HIFU and photodynamic and laser ablation. Notably, not all biochemical recurrences progress similarly. In some patients, disease recurrence remained stable without imaging evidence of distant metastasis, and they died of non-cancer-related causes. Compared with primary therapy, salvage therapy also incurs a higher risk of post-treatment complications, e.g. recto-urethral/vesical fistulas, urethral/bladder neck strictures, haematoma and infection risk and long-term decline in urogenital functionality [34]. Thus, more concrete evidence is needed for the careful selection and counselling of patients with indications and a wide variety of salvage treatment options.

Reasons for Systematic Review

The literature on cryotherapy has shown mixed results regarding its efficacy. In fact, the latest NICE and EAU guidelines recommend cryotherapy only for use in clinical trials due to lack of supportive evidence for use in the clinical setting [35]. Gao et al. have presented a systematic review and meta-analysis of primary and salvage cryotherapy for localised prostate cancer, with inconclusive results in terms of patient survival and oncological outcomes. Moreover, comparisons of treatments are limited to those between radical prostatectomy and radiotherapy. Since the analysis by Gao et al. was published five years ago, further advancements in cryotherapy technology will have likely been made; hence, an updated review is required to consolidate current evidence.

Furthermore, erectile dysfunction is one of the most common complications after cryotherapy [36]. Initial functional outcomes after cryotherapy have been discouraging, given the relatively high rate of erectile dysfunction in up to 95% of patient cohorts. The recent meta-analysis by Zhou et al. has shown that whether focal or whole gland, cryotherapy modalities have shown similar complication rates for erectile dysfunction of up to 40%, which is comparable with that for patients who underwent surgical radical prostatectomy. Other complications, including incontinence and, more severely, urethral or rectal fistulation, are relatively much lower in cryotherapy than in radical prostatectomy. As a shift has been observed from whole-gland cryotherapy to focal target ablation, re-exploring the post-procedural outcomes and preservation of functionality would be worthwhile. Current evidence from systematic reviews of focal therapy compared with more radical treatments remains unclear on its efficacy in terms of disease-specific survival due to poor data quality.

Our primary outcome to identify the recent updates on the oncological and survival outcomes of both primary cryotherapy and salvage cryotherapy. And the secondary outcomes are the updated complication rates and urogenital functional preservation after primary and salvage cryotherapy.

## Review

Methodology

A computer-assisted literature search was performed on the PubMed, Cochrane and Google Scholar databases. The current guidelines from the NICE and EAU were also reviewed. The keywords used included ‘Cryotherapy, Prostate cancer’, ‘Cryoablation, Prostate cancer’ and ‘Cryosurgery, Prostate cancer’. Truncations and Boolean operators were used with the keywords.

Inclusion and exclusion criteria

Studies reviewed included randomised controlled trials and controlled case series studies of patients who underwent cryotherapy. To avoid the overlapping of results with existing systematic reviews with a similar context, the date of research publication was limited to 2015 to July 2021. All included studies had full manuscripts. Non-English research studies were also included, provided that English translations were available.

All studies prior to 2015 and those that did not involve both prostate cancer and cryotherapy were excluded. Studies with only abstracts or no English translation available were excluded, as well.

Data review and extraction

**Figure 1:**
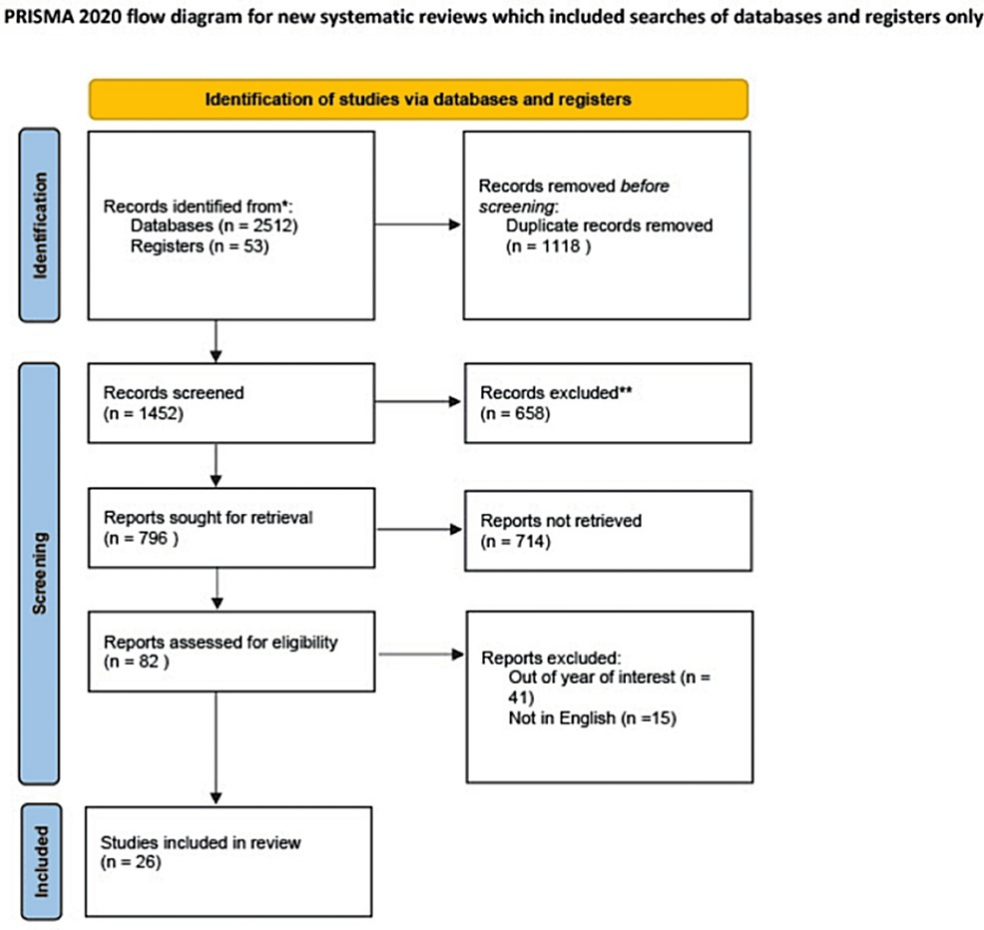
PRISMA review of studies

This review was performed using the Preferred Reporting Items for Systematic Review and Meta-Analysis Guidelines, the process of which is demonstrated in Figure 1. The initial search results retrieved 2,570 studies. Further screening of abstracts eliminated duplicates, and a detailed review of the abstracts of the remaining studies was conducted in view of the research questions, which yielded 82 studies. Further filtering excluded 42 studies without full texts available, 12 that were irrelevant to the primary and secondary outcomes and two without English translations of the full text available.

For each included study, the background of the research was reviewed, and information collected included the year of publication, primary author, number of patient cohorts included, nature of research (whether institutional patient cohorts or patient data extracted from online disease registry databases, e.g., Cryo Online Data and Surveillance, Epidemiology and End Results Programme registries) and mode of study (single arm or double arm; prospective or retrospective). Patient information reviewed also included age, pre-procedural PSA value, prostate biopsy histology results (Gleason score/GG classification), D’Amico risk category and duration of post-procedural follow-up.

In terms of the primary outcome, oncological and survival outcomes can be interpreted variously, including disease-specific survival, overall survival, PSA nadir, biochemical recurrence rate, classification system for defining biochemical recurrence and time of initiating salvage therapy. For the secondary outcomes, complication rate was assessed as number (percentage). For the functional outcomes, the primary focus was on urogenital symptoms. The most common scoring system utilised was the International Prostatic Symptom Score (IPSS) to assess for urinary storage and voiding symptoms. Analysis included assessing the time for a patient to return to baseline score prior to treatment, whereas some studies reviewed the difference between pre- and post-treatment scores. Last but not least, erectile function and sexual functionality were assessed using the International Index of Erectile Function (IIEF) score. All data entry and analysis were performed using the MetaXL software.

Results

Among the 26 studies [23-30,37-54] included (Table 3), two were published in 2016, five in 2017, three in 2018, five in 2019, seven in 2020 and four in 2021. Fifteen cases were single-arm case series studies, whereas 11 studies were double-arm studies. For the double-arm studies, three studies compared the outcome of surgical radical prostatectomy with cryotherapy, two studies compared conservative management with cryotherapy, two studies compared HIFU treatment with cryotherapy, one study compared the treatment modality of cryotherapy with radiological therapy, one study compared modality of cryotherapy with ADT and two studies compared focal/hemi-gland ablation with whole-gland cryotherapy. In terms of patient cohort, there is a total accumulation of 11,228 patients across 26 studies, ranging from a small cohort of 18 patients to 3,051 patients. Four studies of patient cohort were obtained from online registry records. In terms of treatment intention, 11 studies where patients were receiving cryotherapy for salvage approach post failed primary therapy/recurrence disease, remaining 15 studies where cryotherapy was utilised as primary treatment for newly diagnosed prostate cancer.

**Table 3:** Summary of the studies included in the systematic review COLD, Cryo Online Data Registry; HIFU, high-intensity focused ultrasound.

Author/reference	Nature of study	Patient cohort	Patient background
Aminsharifi et al., 2019 [37]	Single-arm retrospective study	108 (91, whole gland; 17, focal)	Salvage indication after failed primary cryotherapy
Bain et al., 2020 [38]	Single-arm retrospective study	58 (37, primary radiotherapy; 21, primary cryotherapy)	Salvage therapy after failed primary therapy
Barat et al., 2019 [39]	Single-arm retrospective study	28 (21, intra-prostatic; 7, extra-prostatic)	Salvage therapy after failed primary therapy
Barqawi et al., 2017 [40]	Single-arm retrospective study	393	Primary treatment
Bauman et al., 2020 [41]	Double-arm retrospective study comparing androgen deprivation therapy and salvage cryotherapy	169	Salvage therapy after failed primary therapy
Bossier et al., 2020 [25]	Double-arm retrospective study comparing whole- and hemi-gland therapies	66 (40, whole gland; 26, hemi-gland)	Primary treatment
Chinenov et al., 2018 [42]	Double-arm retrospective study comparing whole-gland cryotherapy and radical prostatectomy	42	Primary treatment
Chuang et al., 2020 [43]	Single-arm retrospective study	61	Primary treatment
Gestaut et al., 2017 [44]	Double-arm prospective study comparing cryotherapy and brachytherapy	142	Primary treatment
Gevorgyan et al., 2018 [53]	Single-arm retrospective study	97	Salvage cryotherapy
Ginsburg et al., 2017 [30]	Single-arm retrospective study from the COLD registry	898	Salvage cryotherapy
Guo et al., 2020 [23]	Double-arm retrospective study comparing cryotherapy and radical Prostatectomy	1942	Primary treatment
Jin et al., 2020 [24]	Double-arm retrospective study comparing cryotherapy with radical prostatectomy	2350	Primary treatment
Kongnyuy et al., 2017 [45]	Single-arm retrospective study	65	Focal salvage therapy
Lian et al., 2016 [46]	Single-arm retrospective study	32	Focal salvage therapy
Liu et al., 2016 [26]	Double-arm retrospective study comparing cryotherapy and HIFU	114	Primary treatment
Lucan et al., 2017 [47]	Single-arm study	434	Primary treatment
Marra et al., 2021 [27]	Double-arm retrospective study comparing cryotherapy and active surveillance	121	Primary focal treatment
Mercader et al., 2020 [48]	Single-arm retrospective study	177	Primary cryotherapy
Nair et al., 2021 [49]	Double-arm retrospective study comparing cryotherapy and HIFU	186	Salvage recurrent therapy
Oishi et al., 2021 [50]	Single-arm retrospective study	94	Primary cryotherapy
Safavy et al., 2019 [51]	Single-arm retrospective study	75	Salvage therapy post failed primary
Shah et al., 2018 [52]	Double-arm retrospective study comparing conservative management with cryotherapy	3051	Primary cryotherapy
Shah et al., 2019 [28]	Single-arm retrospective studies	122	Primary focal cryotherapy
Tan et al., 2019 [54]	Double-arm retrospective study comparing salvage whole-gland therapy and focal target therapy	385	Salvage cryotherapy
Valerio et al., 2017 [29]	Single-arm retrospective study	18	Primary focal therapy

Primary cryotherapy patient background

For the subsets of primary therapy [23-29,40,42-44,47,48,50,52], the patient age ranged from 55 to 82 years, and pre-procedural PSA ranged from 6.17 to 49.33 ng/mL. For patient prostate biopsy histology, most studies can be divided into three categories corresponding to the D’Amico risk category: Gleason score < 7, Gleason score = 7 and Gleason score > 7. The review included 2,433 patients with Gleason score < 7, 1,958 patients with Gleason score = 7 and 780 patients with Gleason score > 7. In terms of radiological staging, 5,500 patients were stage T1, 2,533 patients were T2 and 75 patients were T3. Lastly, for the D’Amico risk classification, 1,964 patients were considered at low risk, 2,874 were at intermediate risk and 166 were at high risk. The post-treatment follow-up duration ranged widely from 14 to 113 months (Table 4).

**Table 4:** Patient demographics with primary cryotherapy Data are expressed as mean ± standard deviation, median (range), or number (%). ISUP, International Society of Urological Pathology; GS, Gleason score.

Author/reference	Age	PSA (mean/median)	Gleason score	D’Amico	Staging	Follow-up period
Barqawi et al., 2017 [40]	65		6			14
Bossier et al., 2020 [25]	74 (42–81) whole; 76 (71–80) hemi			12, low; 54, intermediate		41 (1.5–99.0) whole gland; 27 (0.9–93.0) hemi-gland
Chinenov et al., 2018 [42]	69 (55–79)	6.5 (4.1–9.1)	21 (GS6), 3 (GS7)			
Chuang et al., 2020 [43]	69 (65–73)	6.6 (4.8–10)	40 (GS7), 15 (GS7), 6 (GS>7)			18 (27 ptn), 6 (31 ptn)
Gestaut et al., 2017 [44]	82	109 (<10), 33 (10–20)	77 (GS6), 65 (GS7)		120 (T1c), 20 (T2a), 2 (T2b)	64.3
Guo et al., 2020 [23]	68.6 (7.4)	6.7(3.3)	939 (GS6), 692 (GS7), 311(>GS8)	805, low; 1137, intermediate	1645 (T1c), 201 (T2a), 96 (T2b)	84 (53,113)
Jin et al., 2020 [24]	68.91 ± 7.55	6.72 ± 3.35	1135 (GS6), 827 (GS7), 388 (GS7)	967, low; 1383, intermediate	1993 (T1), 242 (T2a), 115 (T2b)	
Liu et al., 2016 [26]	69.76 ± 6.49	26 ± 49.33	41 (GS6), 38 (GS7), 36 (>GS8)	19, low; 24, intermediate; 71, high	52 (T3b)	25 ± 7.38
Lucan et al., 2017 [47]	66 ± 6.68	6.17 ± 2.13				
Marra et al., 2021 [27]	66 (62–71)	6.42 (5.03–8.08)	ISUP: 92 (6), 29 (7)	79, low; 40, intermediate; 2, high	101 (T1), 20 (T2)	85 (58–104)
Mercader et al.. 2020 [48]	73.18 (4.8)	8.75 (5.14)	76 (GS7)	57, low; 91, intermediate; 28, high	T1a (117), T2a (47), T2b (10), T2c (3)	60 (32.9)
Oishi et al., 2021 [50]	71 (66–75)	7.5 (5–11)	29 (G6), 49 (G7), 16 (>G7)	25, low; 45, intermediate; 25, high	47 (T1c), 40 (T2), 7 (T3)	67.2 (36–94.8)
Shah et al., 2018 [28]	762 (<69), 70–79 (1919), 370 (>80)				T1 (1425), T2 (1626)	
Shah et al., 2019 [52]	68.7 (64.9–73.8)	10.8 (7.8–15.6)	12 (<7), 108 (7), 2 (>7)	87, intermediate; 35, high	T2 (95), T3 (22)	27.8 (19.5–36.7) months
Valerio et al., 2017 [29]	68 (65–73)	9.54 (5.65–16.00)	G6: 5; G7: 13	13, intermediate; 5, high		

Primary outcomes in primary treatment studies

Ten studies [23,24,27,28,40,43,44,48,50,52] assessed disease-specific survival, which ranged from 90.5% to 100%. Two case series [23,24] compared cancer-specific survival outcomes with radical prostatectomy and obtained hazard ratios ranging from 2.07 to 2.99 with P-values of <0.05. Because of limited comparative double-arm studies and the high heterogeneity of studies, a meta-analysis could not be performed. Five studies [23,27,40,48,52] reported patient overall survival, which ranged from 61.3% to 98.73%. Two studies reported the hazard ratios for comparing the treatment modalities of radical prostatectomy, which ranged from 2.09 to 2.70 with P values of <0.05. Two studies [25,44] assessed biochemical-free survival, which ranged from 53% to 69%. Six studies [26,27,42,44,48,50] reported PSA nadir levels ranging from 0.1 to 2.63 ng/mL. Only one study [21] reported a PSA decrease of 2 ng/mL. Seven studies [25,26,40,44,47,48,50] assessed recurrence rate using the ASTRO Phoenix definition, whereas two studies [27,43] reviewed the rate of positive post-procedural prostate biopsy. The overall recurrence rate ranged from 15.4% to 62% (using the Phoenix definition, from 15.4% to 40.3%), whereas the overall positive recurrence rate by prostate biopsy ranged from 18% to 62% (Table 5).

**Table 5:** Patient oncological outcomes HR, hazard ratio; RP, radical prostatectomy; CI, confidence interval.

Author	Disease-specific survival	Overall survival	Biochemical survival	PSA nadir level	PSA decrease	Recurrence rate	Biochemical recurrence definition
Barqawi et al., 2017 [40]	99.49%	98.73%				20.90%	Phoenix definition
Bossier et al., 2020 [25]			53%–69%			27%	Phoenix definition
Chinenov et al., 2018 [42]				0.62			
Chuang et al., 2020 [43]	Conditional disease-free survival: 98% (6 months); 100% (18 months)				2.0 (0.99–3.5)	18%	Prostate biopsy
Gestaut et al., 2017 [44]	Metastasis-free survival: 97.6%		57.9%	Biochemical failure: 0.8; non-biochemical failure: 0.2		40.30%	Phoenix definition
Guo et al., 2020 [23]	98.1% (10 years); HR compared with RP: 2.07 (1.22–3.51), 95% CI; P = 0.007	61.3% (10 years); HR 2.09 (1.8–2.44), 95% CI; P ≤ 0.001					
Jin et al., 2020 [24]	Mortality: HR 2.99; P = 0.0195	Mortality: HR 2.70; P ≤ 0.0001					
Liu et al., 2016 [26]				0.81 ± 2.29		25.40%	Phoenix definition
Lucan et al., 2017 [47]						15.40%	Phoenix definition
Marra et al., 2021 [27]	100%	97%		2.63 (1.55–3.95)		62%	Positive biopsy
Mercader et al., 2020 [48]	100%	91.50%		0.42 (1.56)		32.70%	Phoenix definition
Oishi et al., 2021 [50]	95%			0.1 (0.0–0.1)		21.30%	Phoenix definition
Shah et al., 2018 [28]	94%						
Shah et al., 2019 [52]	90.50%	96.10%					

Secondary outcomes in primary cryotherapy studies

In terms of post-procedural complications, five studies [25,26,28,29,48] reported urinary incontinence ranging from 1.6.0% to 18.0%. Three studies [26,28,43] reported erectile dysfunction complications, ranging from 3.7% to 88.0%. Seven studies [25,27,29,43,48,50,52] reported urinary retention (4.1% to 18.0%). Four studies [27-29,47] found that urethral rectal fistulas occurred at a rate of 0.82% to 5.50%. In five studies [26-28,40,47] bladder neck stricture/stenosis occurred at 0.83%-13.63%. Nine studies [25-27,29,40,43,47,50,52] reported infections (including UTI and epididymo-orchitis) ranging from 3.0% to 16.7%. Four studies [40,47,48,52] reported complications of chronic pelvic or perineal pain ranging from 2.0% to 9.8%. Six studies [25,27-29,47,48] reported the rate of haematuria developing was from 4.95% to 9.60%. Three studies [25,47,48] reported scrotal/perineal haematoma (6% to 75%), whereas one study [47] reported the rate of developing lower urinary tract symptoms (LUTS) at 34.3%. Finally, one study [28] reported the occurrence rate of hydronephrosis at 2.79%. Table 6 presents the data on post-procedural complications in primary cryotherapy.

**Table 6:** Immediate and long-term complication rates after primary cryotherapy

Author	Incontinence	Erectile Dysfunction	Retention	Fistula	Bladder neck stricture	Infection	Pelvic/perineal pain	Haematuria	Haematoma	LUTS	Hydronephrosis
Barqawi et al., 2017 [40]					9.41%	10.10%	2.04%				
Bossier et al., 2020 [25]	18%		18%			3%		9%	6%		
Chinenov et al., 2018 [42]											
Chuang et al., 2020 [43]		3.70%	7.40%			7.40%					
Gestaut et al., 2017 [44]											
Guo et al., 2020 [23]											
Jin et al., 2020 [24]											
Liu et al., 2016 [26]	1.60%	88%			3.30%	7.30%					
Lucan et al., 2017 [47]				3.60%	3.20%	10%	8.70%	5%	75%	34.30%	
Marra et al., 2021 [ 27]			8.26%	0.83%	0.83%	6.60%		4.95%			
Mercader et al., 2020 [48]	17.50%		8.50%				4.50%	9.60%	11%		
Oishi et al., 2021 [50]			7.44%			6.40%					
Shah et al., 2018 [28]	11%	20.65%		0.82%	13.63%			6.13%			2.79%
Shah et al., 2019 [52]			4.10%			9%	9.80%				
Valerio et al., 2017 [29]	11.10%		16.70%	5.50%		16.70%		5.50%			

Table 7 summarises the functional outcomes after primary cryotherapy. Out of 15 studies, eight described patient functional outcomes. Four studies [26,27,47,50] used the IIEF to compare pre- and post-procedural erectile function outcomes, one study [40] utilised the Sexual Health Inventory for Men questionnaire and one study [43] used the Expanded Prostate Cancer Index Composite for Clinical Practice questionnaire. One study [25] used the clinical definition of erectile dysfunction (number of patients) to assess post-procedural sexual function. Seven studies described continence, five studies [26,27,40,42,43] utilised the IPSS and two studies [25,50] assessed the necessity of requiring continence pads to evaluate urological function. Despite using similar questionnaires, the heterogeneity among the studies was remarkable. In summary, one study found no pre- and post-procedural differences using a questionnaire. Two studies used a sexual function questionnaire to determine post-procedural improvements, whereas three studies showed a post-procedural decline in sexual function. One study indicated differences in pre-and post-procedural sexual outcomes but did not mention either a downward or an upward trend. For continence function, four studies found improved continence symptoms, two studies reported worse continence rates and one study did not mention the trend in the pre- and post-procedural differences.

**Table 7:** Functional outcomes after primary cryotherapy SHIM, Sexual Health Inventory for Men; EPIC-CP, Expanded Prostate Cancer Index Composite for Clinical Practice; IIEF, International Index of Erectile Function; IPSS, International Prostatic Symptom Score.

Author	Sexual function score	Outcomes	Continence scoring	Outcomes
Barqawi et al., 2017 [40]	SHIM pre- and post-procedural differences	4	IPSS pre- and post-procedural differences	3
Bossier et al., 2020 [25]	De novo symptoms	71% pre-procedure, 63% post-procedure	Continence rate	65% (early), 83% (1 year post-procedure)
Chinenov et al., 2018 [42]			IPSS at 12 months	10 (pre), 12 (post)
Chuang et al., 2020 [43]	EPIC-CP (6 months post-operatively)	5–6 insignificant	IPSS	8–5.5 at 18 months
Liu et al., 2016 [26]	IIEF	22.96 ± 2.44 (pre-operatively), 4.18 ± 5.89 (24 months post-operatively)	IPSS	11.73 ± 7.53 (pre-operatively); 9.04 ± 6.30 (24 months post-operatively)
Lucan et al., 2017 [47]	IIEF (3 months)	Pre-operatively: severe, 19.2%; moderate, 19.2%; medium moderate, 36.4%; mild, 18.6%; no, 6.5% Post-operatively: severe, 65.0%; moderate, 14.0%; mild, 4.7%; no, 0.4%		
Marra et al., 2021 [27]	IIEF (3–12 months post-procedure)	10 (pre),14.5 (post)	IPSS	3 (pre), 6 (post)
Oishi et al., 2021 [50]	IIEF (within 2 years post)	36% (pre), 11% (post)	Use of continence pads	98% (pre), 96% (post)

Salvage cryotherapy studies

In 11 studies, 2,101 patients (age range 56-79 years) underwent salvage cryotherapy with the PSA ranging from 0.01 to 31.60 ng/mL. Nine studies [30,37-39,41,45,46,49,54] reported histological staging, and one study [54] reported 7 as the median Gleason score. The remaining studies [51,53] encompassed 536 patients with PSA < 7 ng/mL, 478 patients with PSA = 7 ng/mL and 378 patients with PSA > 7 ng/mL. For the D’Amico risk classification, three studies [37,49,51] included 67 low-risk patients, 121 intermediate-risk patients and 87 high-risk patients. Three studies [30,46,49] reported patient radiological staging, with 222 patients classified as stage T1, 356 patients as T2, 90 patients as T3 and 11 patients as T4. Lastly, patients were followed-up after salvage therapy from 9.0 to 297.6 months. Table 8 presents the patient data obtained from the salvage cryotherapy studies.

**Table 8:** Patient data obtained in salvage cryotherapy studies

Author	Patients involved	Age	PSA (mean)	Gleason score	D’Amico risk	Staging	Follow-up period (months)
Aminsharifi et al., 2019 [37]	108 (91 whole gland; 17 focal)	69.3 ± 7.1	7.08 ± 7.4	GS<7 (43); GS7 (35); GS>7 (25)	L34, M40, H33		43.1 ± 40.8
Bain et al., 2020 [38]	58 (37 primary radiotherapy; 21 primary cryotherapy)	67.2 ± 1 (radiotherapy); 70.8 ± 1.4 (cryotherapy)	6.6 ± 0.6 (radiotherapy); 7.4 ± 0.7 (cryotherapy)	GG1 (10); GG2 (17); GG3 (5); GG4 (19); GG5 (5)			56.1 (radiotherapy), 61.1 (cryotherapy)
Barat et al., 2019 [39]	28 (21 intra-prostatic; 7 extra-prostatic)	69 ± 6	11.5 ± 7.5 intra-prostatic; 17.3 ± 14.3 extra-prostatic	GS<7 (11); GS7 (15); GS>7 (2)			19 ± 10 intra-prostatic; 20 ± 10 extra-prostatic
Bauman et al., 2020 [41]	169	77 ptn (<70 years), 92 ptn (>70 years)	<4 (19); 4–10 (121); >10 (29)	GS<7 (100); GS7 (57); GS>7 (21)			18.65 (17.95–19.90)
Gevorgyan et al., 2018 [53]	97						39.4
Ginsburg et al., 2017 [30]	898	71 (66–76)	5.0 (3.0–8.5)	GS<7 (300); GS7 (279); GS>7 (264)		198 (T1); 273 (T2); 67 (T3); 11 (T4)	19.0 (6.1–51.7)
Kongnyuy et al., 2017 [45]	65	71.0 (65.0–74.3)	4.00 (0.01–19.00)	6 (11); 7 (26); 8 (17)			26.6 (8.0–99.0)
Lian et al., 2016 [46]	32	74 (56–79)	7.9 (3.2–17.6)	6 (8); 7 (9); >7 (15)		4 (T1c); 22 (T2); 6 (T3)	
Nair et al., 2021 [49]	186	67.9 ± 4.4	15 ± 12.6	GS<7 (53); GS7 (35); GS>7 (10)	17 low; 43 intermediate; 38 high	20 (T1); 61 (T2); 17 (T3)	272.4 (256.8–297.6)
Safavy et al., 2019 [51]	75	69.3 (5.98)	18(6), 34(7), 16(>7), Mean 6.0(3.41)		16 low; 38 intermediate; 16 high		46.8 (1.2–114.0)
Tan et al., 2019 [54]	385	70 (66–74)	4.0 (2.7–5.6)	7 (median)			24.4 (9.8–60.3)

Primary outcomes in salvage cryotherapy studies

For oncological outcomes, six studies [37,39,41,46,49,51] reported the cancer-specific survival rate from 65.5% to 100.0%. Two studies [45, 53] reported the range of biochemical-free survival from 48.1% to 58.1%, whereas one study [30] reported an ADT-free survival rate of 71.3%. Three studies [37,46,51] described an overall survival rate of 92.0%-99.1%, whereas two studies [41,49] reported a median survival rate of 11.8-12.33 years. In five studies [37,38,45,46,51], the post-therapy PSA nadir level ranged from 0.01 to 2.0 ng/mL. For biochemical recurrence rate, seven studies [37-39,45,46,51,54] reported a rate of 15.6%-57.5%. All studies defined biochemical recurrence using the Phoenix definition and reported a median follow-up duration until biochemical recurrence of 13.0-74.7 months. Table 9 presents a summary of the primary outcomes in salvage cryotherapy studies.

**Table 9:** Primary outcomes in salvage cryotherapy studies PSA, prostate-specific antigen; PC, prostate cancer; ADT, androgen deprivation therapy.

Author	Disease-specific survival	Overall survival	PSA nadir level	PSA decrease	Recurrence rate	Recurrence criteria	Recurrence duration (months)
Aminsharifi et al., 2019 [37]	91.7%	99.1	0.86 ± 1.73		30.50%	Phoenix definition	
Bain et al., 2020 [38]			0.3 radiotherapy; 2.0 cryotherapy		57.58%	Phoenix definition	18 (PR), 13 (PC)
Barat et al., 2019 [39]	65.50%			5.7 ± 2.6 (intra-prostatic); 6.31 ± 4.5 (extra-prostatic)	33.3%		
Bauman et al., 2020 [41]	83.80%	12.3 3 (years)					
Gevorgyan et al., 2018 [53]	Biochemical-free survival: 58.1%						
Ginsburg et al., 2017 [30]	5-year ADT-free survival: 71.3%						
Kongnyuy et al., 2017 [45]	Biochemical-free survival: 48.1%		0.5 (0.1–1.7)		52.30%	Phoenix definition	
Lian et al., 2016 [46]	100.00%	92.30%	0.20 (0.01–0.60)		15.60%	Phoenix definition	
Nair et al., 2021 [49]	76%	11.8 years (median)					
Safavy et al., 2019 [51]	98.70%	92%	1.40 (3.05)		48.3%	Phoenix definition	22.9 (1.1–74.7) biochemical failure
Tan et al., 2019 [54]					21%		

Secondary outcomes in salvage cryotherapy studies

Table 10 shows the complications in salvage cryotherapy. Six studies [37,39,45,46,53,54] reported urinary incontinence in 6.10%-17.86% of cases. Five studies [37,45,46,53,54] reported erectile dysfunction, ranging from 25.0% to 86.2%. In five studies [37,45,46,51,54], urinary retention complications ranged from 2.13% to 25.3%, whereas four studies [37,51,53,54] reported recto-urethral fistulas in 1.27%-3.7% of cases. Another four studies [38,39,45,51] found that 3.57%-6.67% of patients developed bladder neck strictures, whereas two studies [38,46] found 4.5%-12.5% of patient developed infections. Two studies [39,46] described pelvic perineal pain in 10.71%-31.25% of cases, whereas two case studies [45, 46] reported haematuria at 3.10%-6.25%.

**Table 10:** Complications in salvage cryotherapy

Author	Urinary incontinence	Erectile dysfunction	Urinary retention	Recto-urethral fistula	Bladder neck contracture/stricture	infection	Pelvic/Perineal pain	Haematuria
Aminsharifi et al., 2019 [37]	7.4%	86.2%	3.7%	3.7%				
Bain et al., 2020 [38]					6.10%	4.50%		
Barat et al., 2019 [39]	17.86%				3.57%		10.71%	
Bauman et al., 2020 [41]								
Gevorgyan et al., 2018 [53]	16.46%	83.50%		1.27%				
Ginsburg et al., 2017 [30]								
Kongnyuy et al., 2017 [45]	6.10%	21.50%	4.10%		4.10%			3.10%
Lian et al., 2016 [46]	6.25%	25%	2.13%			12.50%	31.25%	6.25%
Nair et al., 2021 [49]								
Safavy et al., 2019 [51]			25.30%	2.67%	6.67%			
Tan et al., 2019 [54]	14%	57.90%	19.22%	3.38%				

Discussion

This study found that for both primary and salvage cryotherapies, patient characteristics had a fairly good mix in terms of patient Gleason score, D’Amico risk classification and radiologic staging. The cancer-specific, overall and biochemical-free survival rates were relatively similar for both primary and salvage therapies. Erectile dysfunction and urinary retention, followed by incontinence, seemed to be the most common post-procedural complication encountered in both subgroups. Contrary to the hypothesis, the complication rate tended to be higher in the salvage therapy cohort. The complications rate found in this review did not also vary between the subgroups. Functional outcomes were only assessed in primary cryotherapy. Interestingly, some studies found improvements in both continence and sexual function.

The 11 double-arm comparative studies included in this review were heterogeneous in terms of study design, treatment modalities and outcome measures. Thus, a suitable meta-analysis could not be performed to analyse the primary and secondary outcomes, as originally intended. Other treatment modalities compared included active surveillance, radiotherapy and surgical radical prostatectomy. Gao et al. published a meta-analysis and single-arm systematic review in 2016, comparing cryosurgery with other treatment modalities. The pooled data showed similar overall, disease-specific and disease-free survival rates among cryotherapy, radiotherapy and radical prostatectomy. Nonetheless, Gao et al. also showed a high incidence of post-procedural adverse events in cryotherapy. Jung et al. conducted a Cochrane Library-registered meta-analysis and two randomised controlled trials comparing radiotherapy. Unfortunately, the meta-analysis was inconclusive, and no functional outcomes or complications assessment was made because of a lack of available data. Donellay et al. and Bahn et al. assessed long-term oncological and functional outcomes after cryotherapy and found that cryotherapy is comparable with both radiotherapy and radical surgical therapy.

Notably, one of the included studies compared cryotherapy with brachytherapy, which has a similar patient target indication as cryotherapy, essentially for minimally invasive, localised treatment for low- or intermediate-risk patients. Interestingly, the study by Gestaut et al. reported that brachytherapy had a much better biochemical-free survival rate. However, the paper also suggested possible bias in the cryotherapy cohort, which included a generally higher age group. Furthermore, his patients eventually required salvage ADT for recurrence.

The present review showed the range of cancer-specific, overall and biochemical-free survival rates were similar between primary and salvage therapies. However, the data must be interpreted cautiously, as the survival range is relatively wide, and the median follow-up time varied among the studies, which would directly affect the survival assessment.

In terms of recurrence rate, a long-standing debate has persisted on whether the ASTRO or Phoenix definition should be used, which might not be reflective of the actual disease recurrence. This is because the Phoenix definition was originally derived from post-radiotherapy, and yet, somehow, it has been used in outcome assessment for other treatment modalities, as well. Furthermore, repeat PSA measurement is much more easily available and interpreted compared with other means of assessing recurrence. One of the included studies conducted by Barqawi et al. reported that 82 of 392 patients showed positive raised PSA nadir level after primary cryotherapy. Repeat prostate biopsy only showed positive results in 52 patients within the same cohort. This may indicate that both criteria might not be reflective of treatment efficacy. Nonetheless, based on recent studies, the Phoenix definition is still being commonly used regardless of assessing primary or salvage therapy.

In 11 studies included in the present review, the post-procedural PSA nadir ranged from 0.10 to 2.63 ng/mL in primary therapy and 0.01-2.00 ng/mL in salvage therapy. Among these studies, three noted a high post-procedural PSA nadir level of more than 0.2 ng/mL, which is indicative of poor prognosis and a higher rate of treatment failure. Cohen et al. found that approximately 50% of patients managed to achieve a low PSA nadir after primary whole-gland therapy, an effect that persisted across a two-year follow-up.

Another comparison that has been assessed in the past was between focal target therapy and whole-gland therapy. This study was not able to differentiate the subgroups, as the included studies showed a high heterogeneity across patients who underwent both therapies. In one of the included studies, a retrospective study by Bossier et al. compared both the oncological and functional outcomes between partial and whole-gland cryotherapies. Interestingly, not much difference was observed in functional outcome despite the initial indication of partial cryotherapy to primarily preserve as much functionality as possible by salvaging healthy prostatic tissue. Furthermore, the functional outcome of cryotherapy seemed worse compared with external beam radiation therapy, with a greater incidence of urinary dysfunction and sexual dysfunction noted more frequently in cryotherapy. They suggested patient selection may be essential, e.g., for the low-risk group and when multi-parametric MRI indicated hemi/focal gland involvement, focal cryotherapy presents an alternative to more invasive treatment for enhanced preservation of functionality. Marra et al. assessed the long-term treatment outcomes of focal cryotherapy and found that radical treatment for prostate cancer may need to be delayed. However, this does not benefit the overall oncological outcomes, with more than 50% of patients requiring further treatment for disease management.

In previous studies, cryotherapy was primarily offered to patients with mild and, at most, intermediate-risk prostate cancer. Onik et al. found that approximately 50% of patients had intermediate- or high-risk disease, thereby opening up a greater opportunity. The study utilised PSA stability and prostate biopsy to determine cancer recurrence. With the definition, >95% of patients were defined as cancer-free within a median follow-up of 3.6 years. Similarly, the present study included patients considered to be at high risk on the D’Amico classification; nevertheless, the cohort included in this study remained relatively small. Shah et al. compared the outcomes of immediate- to high-risk patients who underwent focal cryotherapy and found no significant differences in relative risk factor or complication rate compared with those of the national cryotherapy registry.

The common side effects or complications of cryotherapy identified in our study included erectile dysfunction, perineal pain, urinary tract infections, urethral strictures, urethral rectal fistulations and voiding dysfunction. Kimura et al. performed a retrospective study of two separate cohorts and found that patients with high pre-operative or pre-procedural IPSS and BI qualitative scores and low uro-flowmetry quantitative scores obtained significantly better scores or outcomes compared with the pre-procedural measurements at 18 months after treatment. They believed cryotherapy possibly caused prostate tissue destruction, which reduced the prostate volume and thus improved functional outcomes, such as urogenital function. Jiang et al. reported a portion of patients complained of voiding necrotic prostate tissue, which caused retention and thus required long-term catheter use [55].

ADT has been utilised primarily for recurrent or metastatic prostate cancer, especially for salvage intent. It not only enables localised targeting but also helps suppress metastatic disease progression. Its non-invasive nature makes it ideal especially for patients not suitable for surgical treatment. Nonetheless, ADT presents risks and side effects, such as low libido, erectile dysfunction, hot flushes, reduced muscle mass, gynaecomastia, testicular atrophy and chronic tiredness, all of which would be poorly tolerated by certain patients. More importantly, as prostate cancer advances, it may become castration-resistant and non-responsive to further ADT. Therefore, local ablative salvage therapy is essential in delaying or preventing the early usage of ADT, not only to reduce side effects but also and more importantly to help delay the occurrence/onset of castration-resistant disease, thereby improving long-term treatment efficacy and suppression of side effects. The results of Bauman et al. from the COLD registry showed that almost all patients eventually developed subclinical metastatic disease requiring ADT. Interestingly, patients who underwent salvage cryotherapy with deferred ADT showed a higher median overall survival rate compared with patients who immediately underwent ADT. However, both cohorts showed similar cancer-specific survival rates.

Strengths and limitations

This is a comprehensive, updated systematic review of the most recent studies on primary and salvage cryotherapies. However, this review found high heterogeneity in study design, treatment modality and assessment of oncological and functional outcomes. This heterogeneity rendered a meta-analysis and the attainment of a statistically significant conclusion impossible. Moreover, some of the included studies did not have all the information required for both primary and secondary analyses. Hence, the available data might not be representative of all patients.

## Conclusions

In conclusion, this systematic review does not find a difference in cancer-specific overall and biochemical-free survival rates comparing primary with salvage cryotherapy cohorts. The commonest complication encountered for both cohorts of the patient included erectile dysfunction, urinary incontinence, lower urinary tract/bladder neck and infection. The patient's functional outcome has mixed results varied from improvement, unchanged and worsening of urogenital function. This review helps supplement and consolidate previous similar reviews and meta-analyses published previously. There is a critical need for more prospective and double-arm studies to help and provide guidance for careful selection of patient cohort for cryotherapy is paramount regardless of curative/salvage intent.
